# Massive Hemothorax Caused by a Single Intercostal Artery Bleed Ten Days after Solitary Minimally Displaced Rib Fracture

**DOI:** 10.1155/2015/120140

**Published:** 2015-11-05

**Authors:** Karleigh R. Curfman, R. Jonathan Robitsek, Gregory G. Salzler, Katherine D. Gray, Charles S. Lapunzina, Ravi K. Kothuru, Sebastian D. Schubl

**Affiliations:** ^1^Ross University School of Medicine, Portsmouth, Dominica; ^2^Department of Surgery, Jamaica Hospital Medical Center, Jamaica, NY 11418, USA; ^3^Department of Surgery, Weill Cornell Medical College, New York, NY 10065, USA

## Abstract

Delayed hemothorax (DHX) following blunt thoracic trauma is a rare occurrence with an extremely variable incidence and time to diagnosis that is generally associated with clinically insignificant blood loss. In this report, we present a case of acute onset DHX ten days after a relatively mild traumatic event that resulted in a single minimally displaced rib fracture. The patient awoke from sleep suddenly with acute onset dyspnea and chest pain and reported to the emergency department (ED). The patient lost over six and a half liters of blood during the first 9 hours of his admission, the largest volume yet reported in the literature for DHX, which was eventually found to be due to a single intercostal artery bleed. Successful management in this case entailed two emergent thoracotomies and placement of multiple thoracostomy tubes to control blood loss. The patient was discharged home on postoperative day 5.

## 1. Introduction

Delayed hemothorax (DHX) is a rare entity with many potential causative factors and an exact definition that has yet to be agreed upon, thus warranting further research. Currently, there is debate over the exact length of time following injury and initial presentation for a hemothorax to be classified as a DHX, as well as differences in the reported incidence of DHX. As per data interpreted by Masuda et al. and produced by Shorr et al., DHX is defined as a hemothorax that develops 24 hours or later following the inciting injury [1, 2]. However, Ritter and Chang considered DHX to occur as early as two hours after presentation when imaging at presentation showed no abnormalities [3]. In one retrospective study spanning two years, 11 of 515 cases of blunt thoracic trauma developed delayed hemothorax, an incidence of 2.1% [2]. In contrast, a study by Misthos et al. reported an incidence of 7.4% over two years, with 52 out of 709 blunt thoracic trauma patients developing delayed hemothorax [4].

Herein we report a unique case of both an exceptionally large and extraordinarily delayed hemothorax, namely, a 6.5-liter acute blood loss that occurred ten days after the inciting incident. There are a limited number of cases with such a delayed presentation and none that had a larger amount of total blood loss. By comparison, the study by Misthos et al. had the largest sample of DHX cases at 52 patients, yet only one had a blood loss greater than 500 mL [4]. Forty-six of the aforementioned 52 DHX patients (88%) presented within the first seven days of the inciting trauma [4], in concordance with the study by Simon et al. where 100% of patients who developed DHX were identified within the first six days following the traumatic event [5]. A PubMed query revealed only five publications on DHX that reported cases presenting at ten days or later, and none had a blood loss of similar volume to that observed in our patient [1, 4, 6–8].

## 2. Case Report

**Figure 1:**
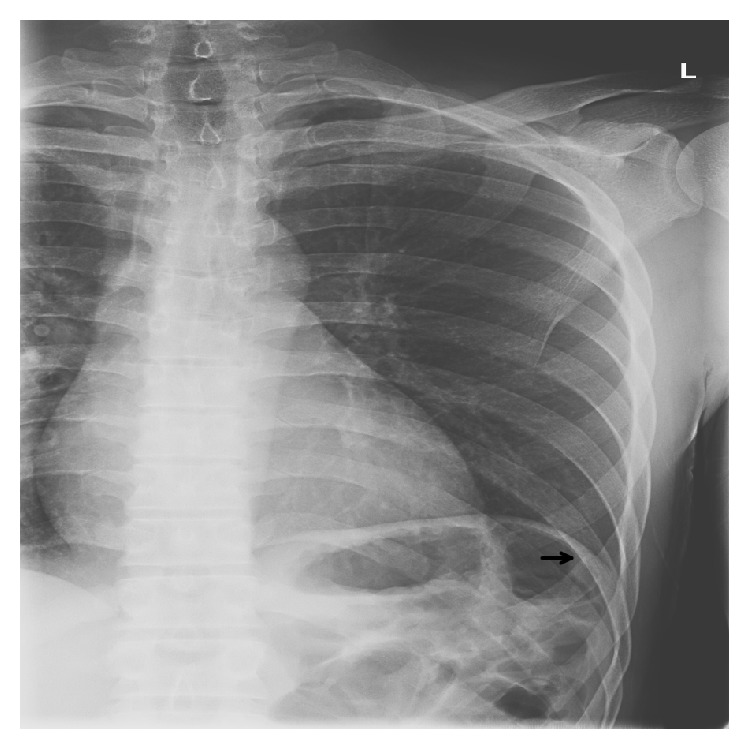
Chest X-ray from the patient's initial ED visit showing anterior lateral left seventh rib fracture (black arrow), with no lung contusion or pleural effusion present.

**Figure 2:**
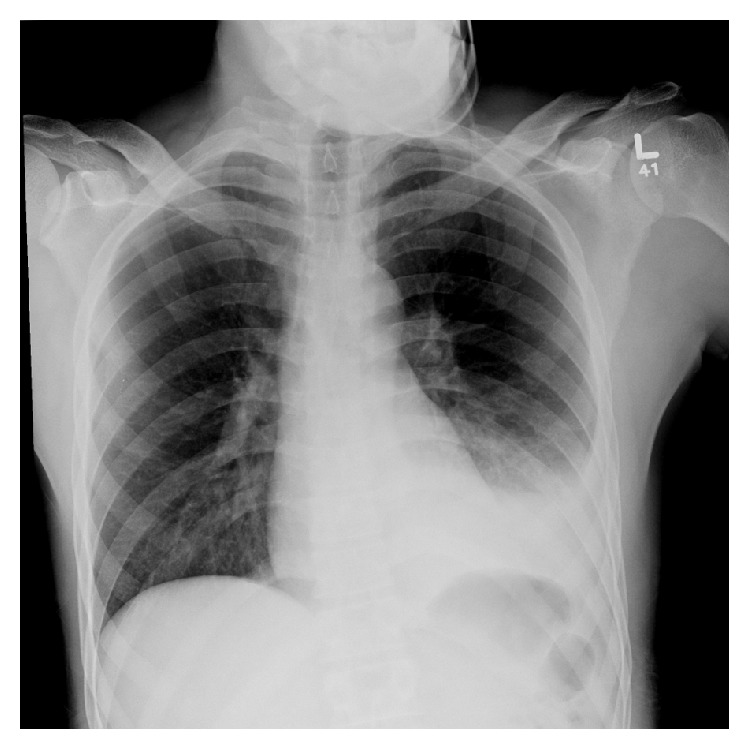
Chest X-ray imaging performed shortly after patient's arrival to emergency department displaying evidence of pleural fluid suspicious of hemothorax in the lower lobe of the left lung.

**Figure 3:**
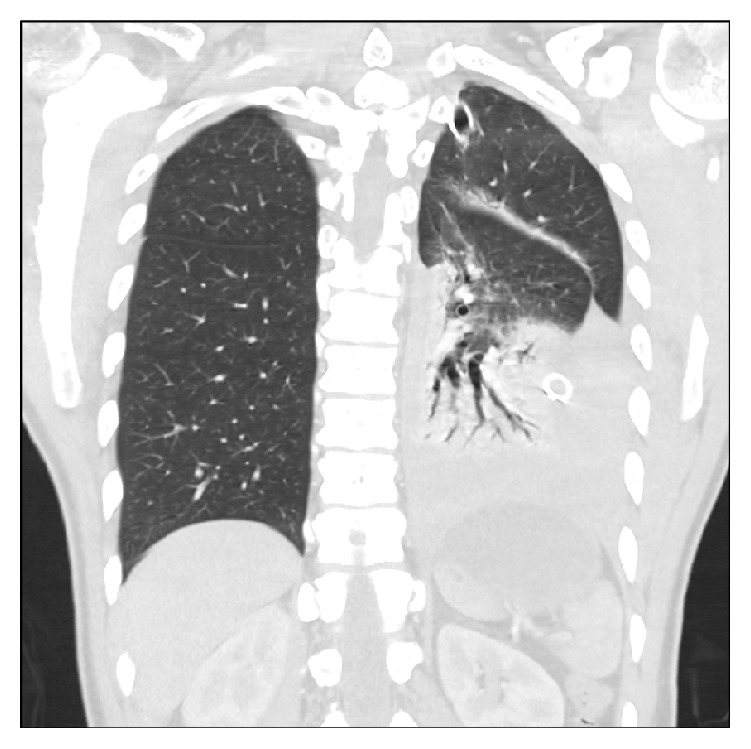
Computed tomography imaging of the chest revealing collapse of the left lower lobe and a large left-sided, high density pleural fluid collection suggestive of hemorrhage continuing into the thoracic cavity despite thoracostomy tube placement.

The patient is a 29-year-old African American male who presented to the emergency department (ED) after being assaulted. This initial presentation to the ED revealed small lacerations to the face and a minimally displaced fracture of the left anterior seventh rib (Figure 1). Further workup found no other serious injuries, personal or family history of bleeding diatheses, connective tissue disorders, or neurofibromatosis and after a total visit time of five hours he was discharged home from the ED with pain medications, incentive spirometry, and proper follow-up instructions. Ten days following initial presentation, the patient returned to the ED with complaints of acute onset severe left sided chest pain, dyspnea, and orthopnea that woke him from sleep with no inciting cause. Initial assessment revealed that the patient was tachycardic, tachypneic, and somnolent. Chest X-ray (CXR) was then performed revealing a large, left sided consolidation suspicious for a hemothorax (Figure 2). A thoracostomy tube was placed immediately yielding 1,700 mL of bright red blood. Subsequently, his mental status deteriorated and he developed severe respiratory distress requiring emergent intubation prior to further evaluation. Computed tomography (CT) imaging revealed collapse of the left lower lobe and a persistent large fluid density on the left side, consistent with hemothorax despite thoracostomy tube drainage and no evidence of a contrast blush (Figure 3). The patient was immediately taken to the operating room (OR) for exploration. A left anterolateral thoracotomy was performed revealing a 1,000 mL blood clot retained in the left chest. Full evaluation of the thorax revealed only areas of abnormal oozing on the visceral pleura that were cauterized as well as a small nonbleeding tear in the diaphragm that was packed with Surgicel. The sites of initial tube thoracostomy as well as the area of the known seventh rib fracture were inspected and no bleeding was noted. A video-assisted thoracoscopic scope (VATS) was inserted through the thoracotomy incision as an adjunct to assist in the evaluation of deep recesses in the pleural cavity which were difficult to visualize directly. The patient received six units of packed red blood cells (PRBC), six units of fresh frozen plasma (FFP), and one unit of platelets during the case, with an intraoperative blood loss of 1,100 mL in addition to the clot already noted. Once double lung ventilation was reestablished, all lobes expanded normally. The patient was transferred to the Surgical Intensive Care Unit (SICU) with two thoracostomy tubes in place. Over the ensuing two hours in the SICU, the patient's chest tubes drained an additional 900 mL of fresh blood necessitating massive transfusion protocol initiation and return to the OR for emergent reexploration of the chest. Upon entry, an additional 1,400 mL of fresh blood was drained from the thorax. The previous operative sites were explored with no active hemorrhage discovered. The intercostal artery at the seventh rib was ligated and several areas of small contusions on the chest wall were explored and cauterized, with no further bleeding noted. During the case, the patient received four additional units each of PRBC and FFP and one unit of platelets, with an additional 250 mL of intraoperative blood loss. Two new thoracostomy tubes were placed, and the patient returned to the SICU in stable condition. The remainder of the patient's hospital course showed a stable hemoglobin and hematocrit with no recurrence of bleed and no other complications. Postoperatively his chest tubes put out a total of 350 mL of additional bloody fluid. He was extubated without incident on postoperative day 1, the apical thoracostomy tube was removed on postoperative day 3, and the basilar thoracostomy tube was removed on postoperative day 4. The patient was discharged home in stable condition on postoperative day 5.

## 3. Discussion

Delayed hemothorax (DHX) has rarely been described. Few retrospective studies exist, and those that do report incidences of DHX ranging from as low as 2.1% up to as high as 7.4% following blunt thoracic trauma, though nearly all of these cases are clinically insignificant [2, 4]. Because of its rarity, there is inconsistency in the definition of what constitutes a “delayed hemothorax.” Some studies used hemothorax that developed after 24 hours with a normal initial CXR as a guideline to define a “delayed” hemothorax [2, 4, 9]. Others used development of hemothorax at any time after initial imaging showing no abnormalities, with or without a confirmatory second CXR [3, 5, 10]. A review of the relatively limited literature revealed that 85% of DHX cases were reported within seven days of the inciting incident, with approximately a quarter of all cases presenting in the first 96 hours [1, 4–13]. Critically, the existing retrospective reviews of DHX define the delay as the time to diagnosis with a follow-up CXR, meaning that the patient's delayed bleed could have occurred at any time prior to that imaging. Since many of these patients are treated on an outpatient basis, this makes the actual time of the delayed bleed unclear. In this case, given his clinical presentation, it seems likely that the patient developed an acute large volume bleed a full ten days after the inciting event, in contrast to the usual slow accumulation of a small volume of blood in the vast majority of previously reported cases.

Individual case series have identified some cases of large volume blood loss in DHX even with significant delay, though large volume blood loss cases are generally early or immediate. The combination of large volume blood loss and DHX is limited to singular cases. Ogawa et al. reported a 2,100 mL blood loss at 30 days following inferior phrenic artery tear [11], Ross and Cordoba reported a 3,500 mL blood loss at under four days [13], and Sharma et al. reported a 2,000 mL blood loss at less than three days [8], with the last two occurring in patients each having four contiguous rib fractures. There have been no prior reports of such a large volume blood loss occurring so late after the initial traumatic event. A life threatening bleed occurring spontaneously in a sleeping patient after relatively mild trauma resulting in just a single minimally displaced rib fracture a full ten days after the inciting trauma is certainly uncommon but a possibility that all trauma and emergency physicians should be aware of.

Due to the paucity of cases of DHX reported, clear guidelines on proper management have yet to be established. The majority of publications opt for more conservative approaches, such as continuous hemodynamic monitoring, thoracocentesis, or thoracostomy prior to progressing to surgery. These recommendations advocate for nonoperative measures in most settings and suggest surgical exploration via thoracoscopy or thoracotomy in instances when the patient is hemodynamically unstable, when there is active or persistent hemorrhage despite attempted drainage, or for clot evacuation [4–6, 9, 10, 12, 13]. Our patient never developed hemodynamic instability despite the 6,350 mL blood loss he suffered in the first 24 hours after he began bleeding, though he did develop significant mental status changes shortly after initial chest tube placement. Our management was concordant with the recommendations for chest trauma in general, and an initial bleed of 1,700 mL of blood with evidence of continued bleeding warranted an emergent thoracotomy. The absence of an obvious source of bleeding at exploration was confounding, as was the immediate need for reexploration based on subsequent continued bleeding. Eventual control of the intercostal vessel and use of topic thrombogenic agents were able to finally staunch his impressive blood loss. Two studies reviewed proposed that using arterial embolization after extravasation of contrast revealed the location of the bleed on CT imaging [7, 11]. This method, though less invasive, is not without potential complications and does not allow for removal of accumulated blood in the chest except by tube thoracostomy, which can only remove unclotted blood and thus potentially can result in a retained hemothorax. One study attempted arterial embolization for a DHX that developed 24 hours following the inciting trauma. However on postembolization day 21, the patient's CT imaging showed extravasation of contrast and a retained hemothorax which ultimately required Video Assisted Thoracic Surgery debridement for definitive management [7]. Another study attempted embolization for DHX one day following the causative trauma; however the patient continued to bleed and was taken for thoracotomy in which the bleed was identified and ligated [11]. Despite our patient's stable hemodynamics, embolization was not an appropriate option given the lack of a blush on CT, known retained blood clot, and degree of blood loss at presentation.

## 4. Conclusion

DHX is a rare complication of chest trauma that can present at a highly variable time interval following the initial traumatic event. Generally, the quantity of blood loss is clinically insignificant and the majority of cases of DHX present within 7 days of the inciting event. The volume of blood lost by the patient presented here is unique as is the acute and persistent nature of his bleed at such a lengthy interval following the causative injury. This case underscores that even patients that have comparatively minor chest trauma days or even weeks earlier are capable of developing life threating bleeding and should be managed in the same manner as acute chest injuries if the clinical presentation is suspicious. Indications for emergent thoracotomy should be no different compared to in trauma patients that present at the time of injury, though there are options for noninvasive angiographic techniques in stable patients. These, however, should be carefully considered as they have been reported to be of variable efficacy and are not capable of clearing the chest cavity of retained blood beyond what can be mobilized by tube thoracostomy. The type of thoracotomy performed should be dictated by the injuries identified on preoperative imaging, as is the case in acute chest trauma. However, if the source of blood loss is not clear preoperatively, then an anterolateral approach is generally recommended as it provides the broadest exposure. Though VATS is being increasingly used at specialized trauma centers for thoracic injury, we feel that an open thoracotomy is the most expeditious approach for managing these patients, particularly in the setting of massive blood loss or hemodynamic instability, as VATS has been reported to be most effective and without significant complication within 24 hours of injury [14].

## References

[1] Masuda R., Ikoma Y., Oiwa K., Nakazato K., Takeichi H., Iwazaki M. (2013). Delayed hemothorax superimposed on extrapleural hematoma after blunt chest injury: a case report. *Tokai Journal of Experimental and Clinical Medicine*.

[2] Shorr R. M., Crittenden M., Indeck M., Hartunian S. L., Rodriguez A. (1987). Blunt thoracic trauma analysis of 515 patients. *Annals of Surgery*.

[3] Ritter D. C., Chang F. C. (1995). Delayed hemothorax resulting from stab wounds to the internal mammary artery. *The Journal of Trauma*.

[4] Misthos P., Kakaris S., Sepsas E., Athanassiadi K., Skottis I. (2004). A prospective analysis of occult pneumothorax, delayed pneumothorax and delayed hemothorax after minor blunt thoracic trauma. *European Journal of Cardio-Thoracic Surgery*.

[5] Simon B. J., Chu Q., Emhoff T. A., Fiallo V. M., Lee K. F. (1998). Delayed hemothorax after blunt thoracic trauma: an uncemmon entity with significant morbidity. *Journal of Trauma: Injury, Infection and Critical Care*.

[6] Khoschnau S. R., Tuma M. A. M., Maull K. (2012). Delayed post-traumatic hemothorax. *Journal of Emergency Medicine, Trauma and Acute Care*.

[7] Yamanashi K., Nakao S., Idoguchi K., Matsuoka T. (2015). A case of delayed hemothorax with an inferior phrenic artery injury detected and treated endovascularly. *Clinical Case Reports*.

[8] Sharma O. P., Hagler S., Oswanski M. F. (2005). Prevalence of delayed hemothorax in blunt thoracic trauma. *The American Surgeon*.

[9] McLoughlin R., Mulcahy R., Kent P., Al-Delamie T., Aherne T. (1987). Haemothorax after rib fracture—incidence, timing and prediction. *Irish Journal of Medical Science*.

[10] Chinnan N. K., Shabaan A. I. M., Palkar S. D. (2006). Delayed life-threatening hemothorax without rib fractures after blunt chest trauma. *Indian Journal of Critical Care Medicine*.

[11] Ogawa F., Naito M., Iyoda A., Satoh Y. (2013). Report of a rare case: occult hemothorax due to blunt trauma without obvious injury to other organs. *Journal of Cardiothoracic Surgery*.

[12] Plurad D., Green D. J. (2008). Diaphragmatic injury presenting as delayed hemothorax. *Military Medicine*.

[13] Ross R. M., Cordoba A. (1986). Delayed life-threatening hemothorax associated with rib fractures. *Journal of Trauma*.

[14] Goodman M., Lewis J., Guitron J., Reed M., Pritts T., Starnes S. (2013). Video-assisted thoracoscopic surgery for acute thoracic trauma. *Journal of Emergencies, Trauma and Shock*.

